# Soliton-like coherent structures: a key to opening the door to turbulence

**DOI:** 10.1093/nsr/nwaf535

**Published:** 2025-11-27

**Authors:** Shiyi Chen, Xiaogang Deng, Cunbiao Lee

**Affiliations:** State Key Laboratory for Turbulence and Complex Systems, School of Mechanics and Engineering Science, Peking University, Beijing 100871, China; School of Mechanical Engineering and Mechanics, Eastern University of Science and Technology, Ningbo, Ningbo 315200, China; Chinese Academy of Military Science, Beijing 100071, China; National Key Laboratory of Fundamental Algorithms and Models for Engineering Simulation, Sichuan University, Chengdu 610207, China; State Key Laboratory for Turbulence and Complex Systems, School of Mechanics and Engineering Science, Peking University, Beijing 100871, China

**Keywords:** soliton-like coherent structure, origin of turbulence

## Abstract

This review summarizes recent advances in the origin of turbulence. The soliton-like coherent structure (SCS) has been identified not only as a common structure in natural and K-type transitions, but also as a fundamental structure in N-type, O-type and bypass-type transitions, which dominate turbulence generation. It has also been confirmed as an essential structure in turbulent boundary layers. Notably, the SCS has been successively observed in pipe flows, stratified flows, mixing layers, jet flows, falling films and wakes. These findings collectively indicate that the SCS serves as a fundamental structure in shear flows, providing significant insights into the origin of shear turbulence.

## INTRODUCTION

The core objective of this paper is to present certain experimental facts and physical structures obtained under advanced data-processing conditions, with the aim of assisting theoretical researchers in refining past models, correcting potentially flawed ones and establishing new physical models based on these findings. This paper addresses three specific aspects.

First, it summarizes the existence of the soliton-like coherent structure (SCS) in the boundary layer under various inflow conditions in recent years, particularly since 2020. This fundamentally reveals a concise and unified physical process under complex inflow conditions, which is beneficial for future theoretical calculations, experimental studies and even the development of turbulence models.

Second, it synthesizes recent findings demonstrating that the SCS remains universally present in a broad class of shear flows. This indicates that the research methodologies for the SCS in boundary layers can be extended to other shear flows, and further suggests that shear flows share the same turbulence generation mechanisms.

Third, it provides feasible methods, research ideas and solutions for the study of turbulence origins. In particular, it offers readers new data-processing techniques that enable the extraction of physically meaningful structures from three-dimensional spatiotemporal data, rather than the previously mathematically defined flow structures. These earlier flow structures have, to varying degrees, led to misunderstandings and, in some cases, consumed substantial research effort while failing to yield authentic physical insights—most notably in approaches relying on timelines and material surfaces. The methodologies proposed here merit broad application in future investigations.

The contributions of this paper are three-fold. First, it demonstrates that the SCS is a common physical structure underlying the origin of shear turbulence. After 30 years of effort, particularly since 2020, the SCS has been shown to play a decisive role in the origin of turbulence in boundary layers, stratified flows, wake flows, falling flows, mixing layers and jet flows.

Second, it provides a mathematical definition and physical description of the SCS, further clarifying that the SCS is a localized three-dimensional wave structure generated by the interaction of two three-dimensional oblique waves, involving both linear and nonlinear interactions. Previous studies suggested that the interaction of two three-dimensional oblique waves directly leads to the formation of low-speed streaks, overlooking the existence of the SCS. This oversight complicated the research and made it difficult to capture the essential physics.

Third, the study effectively bridges key historical achievements in turbulence research with the discovery of the SCS. It postulates that the SCS governs the turbulent bursting process, the acknowledged mechanism underlying turbulence generation. In contrast to traveling waves, the SCS is therefore identified to be the fundamental building block.

## DEFINITION OF THE SCS

Turbulence is among the most enduring challenges in classical physics and one of the seven Millennium Prize Problems in mathematics. A systematic summary of turbulence generation can be found in our earlier review articles [1,2]. Readers may refer to these papers or earlier literature located through them. Without further ado, this article will directly focus on the role of the SCS, particularly research progress in the study of the SCS since 2020.

In fluid mechanics, it is challenging to provide a strict definition of a flow structure. However, a clearer definition of a wave in the flow can be found in Lighhill’s book [3]. Coherent structures—loosely defined as regions with concentrated vorticity, characteristic and flow-specific organization, recurrent patterns, appreciable lifetime and scale—have long been the primary subject of scientific curiosity and debate in turbulence [4]. Flow visualization has revealed that turbulence, once considered a random phenomenon, inherently contains distinctly ordered structures. These coherent structures can be categorized through flow visualization, such as Hama’s $\Lambda$ vortices, which play a significant role in transition, Kachanov’s chain of ring vortices and Kline’s low-speed streaks, among others. Most of these structures lack rigorous mathematical or physical definitions. To help readers better understand this concept, we provide references to the relevant literature [5–7].

Over the past three decades, the research community’s understanding of the SCS has evolved gradually, leading to a comprehensive understanding. Coupled with recent progress in the authors’ research on receptivity experiments [8–10], defining the SCS has become feasible. Before providing a definition of the SCS, let us summarize its physical characteristics.

1. It is a localized three-dimensional wave.
2. Its convection velocity is generally lower than the mainstream flow velocity, typically about 60%–80% of the incoming flow velocity.
3. Its amplitude depends on the magnitude of the incoming flow disturbances. In cases of small incoming flow disturbances, it manifests as a linear three-dimensional wave; with larger incoming flow disturbances, it typically appears as a nonlinear wave.
4. Its frequency is roughly consistent with the frequency predicted by linear stability theory. After its formation, it amplifies and evolves in the shear layer before gradually diminishing. Because of its small shear gradient, it generally has a longer lifetime before dissipating. This is a key reason why we refer to it as the SCS. It shares certain properties with a soliton, but it is evolving and not a soliton in the mathematical sense.
5. Previously, the SCS was believed to arise from the interaction of two oblique waves, either linear or nonlinear. Recent experimental evidence has confirmed that the SCS is indeed generated by the interaction of two oblique waves.
6. The SCS is distinct from the three-dimensional Tollmien–Schlichting (T–S) wave packet.

Thus, we can define the SCS as a localized three-dimensional wave generated by the interaction of two three-dimensional oblique waves of identical frequency. In the flow direction, it convects downstream at a speed lower than the mean flow. Its frequency is close to the frequency predicted by linear stability theory. Its amplitude first grows to saturation and then diminishes. The SCS has a longer lifetime than other coherent structures, exhibiting properties similar to solitons, but undergoes evolution and dissipation.

The two-oblique-wave interaction can be described as follows. The linear solution form for a pair of three-dimensional oblique waves is


(1) \begin{eqnarray*}
&& A(x,y,z,t)= \phi _{+}(\varepsilon x,y)\exp (\mathrm{i}\alpha x+\mathrm{i}\beta z-\mathrm{i}\omega t) \\
&&\qquad +\, \phi _{-}(\varepsilon x,y)\exp (\mathrm{i}\alpha x-\mathrm{i}\beta z-\mathrm{i}\omega t) \\
&&\qquad +\, \mathrm{c.c.},\quad \varepsilon \ll 1,
\end{eqnarray*}

where $\phi _{+/-}=(u,v,p,T,w)^T $ is a column vector consisting of five variables, with its specific form dependent on the characteristics of the flow field. The variables $\alpha$, $\beta$ and $\omega$ are the streamwise wave number, spanwise wave number and frequency, respectively. The variables *x, y, z* and *t* are the streamwise coordinate, the wall-normal coordinate, the spanwise coordinate and time, respectively. The variables *u, v, w, p* and *T* denote the velocities in the streamwise, wall-normal and spanwise directions, pressure and temperature, respectively. Typically, it adopts a modal-solution format (solving the Orr–Sommerfeld equation or parabolic stability equation) or the optimal disturbance format (solving the transient growth equation). The term c.c. denotes the complex conjugate.

The weakly nonlinear solution form for a pair of three-dimensional oblique waves (retaining only second-order terms) is


(2) \begin{eqnarray*}
&& A(x,y,z,t)=\varepsilon \phi _{+}(\varepsilon x,y)\exp (\mathrm{i}\alpha x+\mathrm{i}\beta z-\mathrm{i}\omega t) \\
&&\quad +\,\varepsilon \phi _{-}(\varepsilon x,y)\exp (\mathrm{i}\alpha x-\mathrm{i}\beta z-\mathrm{i}\omega t) \\
&&\quad +\, \varepsilon ^2\phi _{00}(\varepsilon x,y) \\
&&\quad +\, \varepsilon ^2\phi _{22+}(\varepsilon x,y)\exp (2\mathrm{i}\alpha x+2\mathrm{i}\beta z-2\mathrm{i}\omega t) \\
&&\quad +\, \varepsilon ^2\phi _{22-}(\varepsilon x,y)\exp (2\mathrm{i}\alpha x-2\mathrm{i}\beta z-2\mathrm{i}\omega t) \\
&&\quad +\, \varepsilon ^2\phi _{02}(\varepsilon x,y)\exp (2\mathrm{i}\beta z) \\
&&\quad +\, \varepsilon ^2\phi _{20}(\varepsilon x,y)\exp (2\mathrm{i}\alpha x-2\mathrm{i}\omega t) \\
&&\quad +\, O(\varepsilon ^3)+\mathrm{c.c.},
\end{eqnarray*}

where $\phi _{00}$ represents the mean flow distortion, $\phi _{22}$ and $\phi _{20}$ correspond to harmonic waves, and $\phi _{02}$ denotes the streak disturbance. These higher-order terms are typically obtained by solving the nonlinear parabolic stability equations (NPSEs).

**Figure 1.**
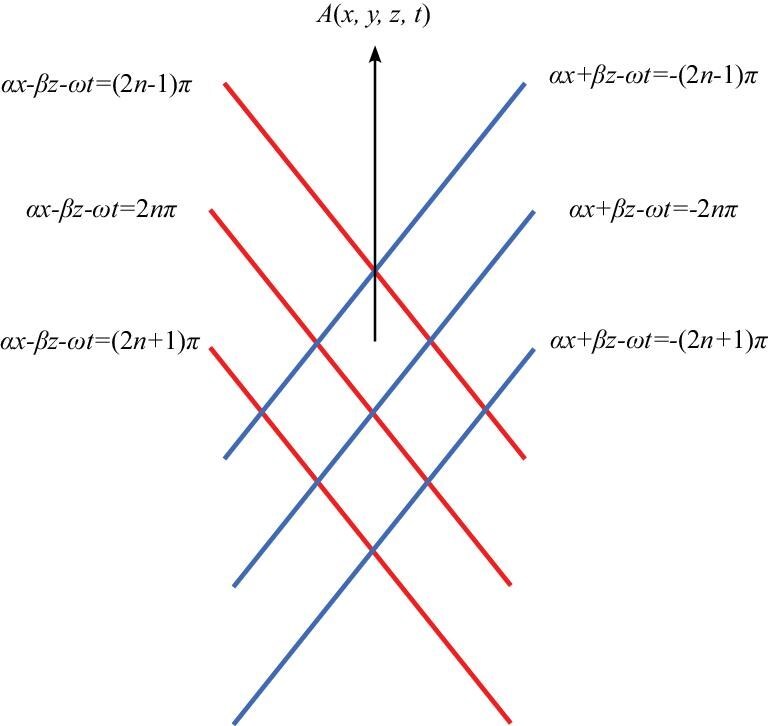
The diamond-shaped structure of the perturbation field.

Specifically, in the case of linear disturbances, the perturbation field exhibits a diamond-shaped structure, with its boundaries delineated by the phase lines shown in Fig. 1, where *n* is an integer.

To confine the disturbances within the ‘diamond-shaped’ region, the following two approaches can be employed.

*Method 1.* Introduce a limiter to ensure that the disturbances are nonzero only within the diamond-shaped region:


(3) \begin{eqnarray*}
{\tilde A}(x,y,z,t)=F(x,z,t)A(x,y,z,t).
\end{eqnarray*}

Here


\begin{eqnarray*}
&&F(x,z,t) = [H(\alpha x+\beta z-\omega t+2n\pi ) \\
&&\qquad -\, H(\alpha x+\beta z-\omega t+2(n-1)\pi )] \\
&&\qquad \times \, [H(\alpha x-\beta z-\omega t-2(n-1)\pi ) \\
&&\qquad -\, H(\alpha x-\beta z-\omega t-2n\pi )]
\end{eqnarray*}

and *H* is the Heaviside function:


\begin{eqnarray*}
H(x)=\left\lbrace \begin{array}{@{}l@{\quad }l@{}}1&\quad \text{for } x>0, \\
0&\quad \text{for } x\le 0. \end{array}\right.
\end{eqnarray*}

The limiter can also be constructed using smoother functional forms, such as the tanh function, while maintaining a similar principle.

*Method 2.* Superimpose multiple disturbance waves with similar wave numbers:


(4) \begin{eqnarray*}
{\tilde A}(x,y,z,t)&=& \int _{\alpha _{0}-\Delta }^{\alpha _{0}+\Delta } \int _{\beta _{0}-\Delta }^{\beta _{0}+\Delta }c_{\alpha \beta }\phi _{\alpha \beta }(y) \\
&&\times \exp (\mathrm{i}\alpha x+\mathrm{i}\beta z-\mathrm{i}\omega t)\mathrm{d}\alpha \mathrm{d}\beta.\\
\end{eqnarray*}

Here $c_{\alpha \beta }$ involves an undetermined constant that depends solely on the wave number. The purpose of selecting this constant is to ensure that the disturbance’s peak aligns precisely with the designated diamond-shaped region.

The two methods each have their own advantages and disadvantages. The primary advantage of method 1 is its simplicity of construction, as it does not involve any undetermined parameters. Additionally, the base solution $A(x,y,z,t)$ can be either a linear or weakly nonlinear solution. However, a significant drawback is that multiplying the solution by the limiter causes it to no longer satisfy the disturbance equations. In contrast, method 2 retains the advantage of satisfying the linear disturbance equations. Nonetheless, it is relatively more complex to construct and requires the determination of undetermined constants, which can increase its complexity.

## DIFFERENCES BETWEEN THE SCS OF LEE AND THE CS SOLITONS OF KACHANOV

The naming of the SCS has a rather intricate backstory, tracing back to C. B. Lee’s participation in the 1993 IUTAM Symposium on Non-Parallel Flow Instability [11]. At the conference, Y. S. Kachanov presented his findings on CS solitons, which he later published in his seminal work in the *Annual Review of Fluid Mechanics* [6]. As young scholars, we were profoundly inspired and resolved to visualize Kachanov’s CS solitons through flow visualization techniques upon our return. Remarkably, we discovered what is now referred to as the SCS. At the time, we genuinely believed we had successfully visualized Kachanov’s CS solitons.

Subsequently, we presented this work at the IUTAM Symposium held in Manchester, UK, in 1995 [12], later published in *Physics Letters A* [13]. We were confident that it confirmed Kachanov’s discovery. However, Kachanov, whose work was based on one-dimensional hot-wire measurements, contested the equivalence of our SCS with his CS solitons. This disagreement was deeply disappointing for us. Nonetheless, Kachanov suggested investigating whether the SCS observed in K-type transition experiments could indeed be related to his CS solitons. This marked the beginning of a collaboration between China and Russia.

There are three landmark experimental works in the K regime of boundary-layer transition research. The first was Klebanoff’s pioneering work [14], which, for the first time in human history, artificially generated turbulence—historically known as the K regime of boundary-layer transition. The second milestone work was accomplished by Hama, who discovered the $\Lambda$ vortex, highlighting the importance of three-dimensional structure generation in transition research [5]. The third milestone work was Kachanov’s experiment [6], where he found that the breakdown of the $\Lambda$ vortex remains periodic. He discovered that the $\Lambda$ vortex generated four high-frequency vortices, termed a chain of ring vortices, providing the first solid experimental data in human history demonstrating the evolution of flow structures from low frequency to high frequency during transition. Were it not for the advancements in modern flow visualization, measurement techniques and data-processing methods, their work might well have concluded research on K-type transition.

Later, the K-type transition experiments conducted at Peking University confirmed that Kachanov was correct. The CS solitons identified by Kachanov [6] were not the same as the SCS we reported at the IUTAM Symposium [12]. We subsequently published a series of papers clarifying the distinctions between the SCS identified by us and the CS solitons described by Kachanov. We have long praised Kachanov’s groundbreaking discovery, but growing experimental and computational evidence suggests that the SCS we identified dominates turbulence generation. We refrain from elaborating on its importance here.

Table 1 summarizes the differences between Kachanov’s CS solitons and Lee’s SCS. The shared nomenclature between Lee’s SCS and Kachanov’s CS solitons originated from a misunderstanding of Lee [12,13]. When we began natural transition experiments in 1993, we read Kachanov’s literature, but failed to fully grasp its depth. Moreover, Kachanov’s key results, derived from one-dimensional hot-wire data, were challenging to interpret. When Lee observed three-dimensional waves during natural transition experiments, he assumed, perhaps too simplistically, that he had visualized Kachanov’s CS solitons. However, subsequent findings proved otherwise, leading to significant misinterpretations among later researchers citing this work.

**Table 1.** Differences between the CS solitons discovered by Kachanov and the SCS discovered by Lee.

Kachanov’s CS soliton [6,15,16]	Lee’s SCS [1,17]
(1) CS solitons are vortex structures; see (i).	(1) Lee’s SCS is a wave structure; see (v).
(2) CS solitons originate in the outer layer of the boundary layer, during the late stages of transition ($x = 500$ mm). They are high-frequency vorticesformed by the stretching and reconnection of $\Lambda$ vortices; see (ii).	(2) Lee’s SCS emerges during the early stages of the transition ($x \le 260$ mm), extending from the lower to the outer layer of the boundary layer and preceding the appearance of Kachanov’s CS solitons; see (ii) and (vi).
(3) After 2000, Kachanov referred to the CS soliton as a ‘ring-like vortex’ (see (iii)).	(3) Lee’s SCS is generated by the interaction of two oblique waves, which give rise to $\Lambda$ vortices and secondary vortex rings. The interaction between the $\Lambda$ vortices and secondary vortex rings results in the formation of Kachanov’s CS solitons, i.e. the ring-like vortex; see (ii) and (vii).
(4) CS solitons are high-frequency structures (relative to $\Lambda$ vortices); see (iv).	(4) Lee’s SCS shares the same frequency as the $\Lambda$ vortices, while Kachanov’s CS soliton is a high-frequency structure; see (iv) and (v).

The main characteristics of Kachanov’s CS solitons are as follows.

- *CS solitons are vortex structures.* As stated in Kachanov’s famous paper [6] on page 462, ‘Thus, experimental studies (which correlate very well with numerical experiments by Rist 1990, 1992, and private communication) show that the CS-solitons at the 3D stages of their development represent very localized (toroidal, most probably) vortices, observed in oscilloscope traces as a set of spikes.’
- *CS solitons appear at the late stage of transition, and they are generated by multiple reconnections of the $\Lambda$ vortex ‘legs’ near its tip and the formation of ring-like vortices*. Kachanov [6] also pointed out on page 468 that ‘The most typical features of the CS-solitons are the spikes observed near the external edge of the boundary layer. These solitons correspond to typical eddies, i.e. the coherent structures observed in the outer part of the turbulent boundary layer’, and claimed on page 460 that, ‘Comparison of these graphs with those obtained for the stage where the soliton has just formed visually demonstrates the wonderfully strong conservatism of the CS-soliton properties mentioned above.’ Bake *et al.* [16] believed that (page 2) the CS solitons were generated by ‘multiple reconnections of the $\Lambda$-vortex “legs” near its tip and formation of the ring-like vortices (through an intermediate stage of the $\Omega$-vortices, or the hair-pin vortices) which represent very stable, soliton-like coherent structures (called the CS-solitons). They have very conservative (slow dissipation) properties and are closely connected with the spikes.’
- *CS solitons are also called ring-like vortices.* Bake *et al.* [16] proposed that (page 18) ‘these positive spikes are discussed in Kachanov [12], Borodulin and Kachanov [17] and corresponds to a positive velocity fluctuation around the external edge of the ring-like vortex (the CS-soliton) attributed to the spike at late stages of K-regime of transition.’
- *Kachanov believed that CS solitons were high-frequency structures compared with the $\Lambda$ vortices.* Kachanov [6] concluded that (page 454) ‘the eventual localization of high-frequency disturbances ultimately led to the soliton formation observed experimentally.’
  The main characteristics of Lee’s SCS are as follows.
- *The SCS is the real three-dimensional wave instead of a vortex*. In Lee [17] on page 3662, ‘The SCS suggested by Lee, which are different from those observed by Kachanov (1994), are typically three-dimensional wave packets, although they have the same frequency as the TS waves.’ Lee and Wu [1] further emphasized that (page 4) ‘First, it is really a wave because it is an oscillating disturbance propagating downstream at a finite speed. Second, a SCS is a nonlinear wave as exhibited by its pattern shown in Fig. 4, where the amplitude develops to a strong localized pattern very quickly in the initial nonlinear stage, and obviously decreases in the later stage of boundary-layer transition. Third, a SCS is a nonlinear traveling wave rather than an ordinary vortex because it keeps irrotational from its birth to death.’
- *The SCS appears at the initial stage of transition before $x=260$ mm from the leading edge and occupies boundary-layer thickness from the near-wall region to the outer layer, with the longest lifetime compared with other coherent structures*. Lee [17] showed that (page 3666) ‘both the visual results and experimental measurement demonstrate the existence of the CS solitons from the initial stage of three-dimensional structure formation ($x = 260$ mm) to the later stage of transition and the onset of turbulence. According to the definition of Kachanov and his co-workers [8], the CS solitons they observed are the outer part of the chain of ringlike vortices.’ CS solitons are present not only in the near-wall region, but also near the external part of the boundary layer. The main features of CS solitons remain unchanged before breakdown, which is why they are called CS solitons.
- *The SCS is formed by the interaction of two oblique waves; it induces the formation of the $\Lambda$ vortex and a secondary closed vortex, and the interaction between the $\Lambda$ vortex and the secondary closed vortex generates a chain of ring vortices, i.e. Kachanov’s CS solitons.* Lee and Wu [1] clearly showed that (page 8) ‘The physical mechanism for the SCS generation and its relation with the low-speed streak is that a rhombus SCS comes from the nonlinear interactions between two oblique waves generated at the leading edge of the flat plate.’ All the dynamic processes for the formation of the secondary closed vortex and a chain of ring vortices are clearly shown in the related part of the paper by Lee and Wu [1].

## SCS: THE COMMON FLOW STRUCTURE IN THE BOUNDARY LAYER DOMINATING TURBULENCE PRODUCTION

Morkovin *et al.* [18] summarized the transition paths based on the magnitude of disturbances (see fig. 1 of [19]). When the incoming disturbance is relatively weak, the boundary-layer receptivity undergoes modal linear growth and nonlinear-interaction processes, eventually breaking down into turbulence. This process is referred to as natural transition. Depending on the different mechanisms of the nonlinear interaction, natural transition includes K-type transition, N-type transition (including C-type and H-type) and O-type transition. When the incoming disturbance is relatively strong, the transition process in which disturbances bypass the linear-growth stage and enter directly into the nonlinear-interaction stage is referred to as bypass transition.

The K-type transition, first discovered experimentally by Klebanoff *et al.* [14], is generally described as wave resonance. The K-type transition involves the interaction of a two-dimensional primary wave $(\omega _1, 0)$ with a pair of oblique waves with the same frequency as the primary wave $(\omega _1, \pm \beta )$. Alternatively, it can be represented as the Craik–Nayfeh–Bozatli four-wave resonance [6], which involves the interaction of a two-dimensional primary wave $(\omega _1, 0)$, a pair of oblique waves of identical frequency $(\omega _1, \pm \beta )$ and a two-dimensional wave with twice the primary wave frequency $(2\omega _1, 0)$. The K-type transition can be expressed using the following equations, where the coordinate axes *x, y* and *z* represent the streamwise, wall-normal and spanwise directions, respectively:


(5) \begin{eqnarray*}
\left\{ \begin{array}{@{}l@{}}
f_{1} = \widehat{A_{1}(y)}\exp (\mathrm{i}\alpha x - \mathrm{i}\omega t), \\
f_{2} = \widehat{A_{2}(y)}\exp (\mathrm{i}\alpha x + \mathrm{i}\beta z - \mathrm{i}\omega t),\\
f_{3} = \widehat{A_{3}(y)}\exp (\mathrm{i}\alpha x - \mathrm{i}\beta z - \mathrm{i}\omega t).
\end{array} \right.
\end{eqnarray*}

The N-type transition primarily refers to the transition triggered by subharmonic resonance, which involves the resonance of a two-dimensional wave $(\omega _1, 0)$ and a pair of oblique waves $(\omega _{1/2}, \pm \beta )$ with identical wave angles but opposite directions. The frequency of the oblique waves is half that of the two-dimensional wave. Both the three-wave resonance mechanism proposed by Craik [20] (C-type transition) and the secondary instability mechanism proposed by Herbert [21] (H-type transition) are categorized as N-type transitions. The main difference between C-type and H-type transitions lies in the wave angle requirements, with H-type transitions having more relaxed constraints.

The C-type transition can be expressed using the following equations:


(6) \begin{eqnarray*}
\left\{ \begin{array}{@{}l@{}}
f_{1} = \widehat{A_{1}(y)}\exp (\mathrm{i}\alpha _{1}x - \mathrm{i}\omega _{1}t), \\
f_{2} = \widehat{A_{2}(y)}\exp (\mathrm{i}\alpha _{2}x + \mathrm{i}\beta _{2}z - \mathrm{i}\omega _{2}t), \\
f_{3} = \widehat{A_{3}(y)}\exp (\mathrm{i}\alpha _{3}x - \mathrm{i}\beta _{3}z - \mathrm{i}\omega _{3}t).
\end{array} \right.
\end{eqnarray*}

Here



\begin{eqnarray*}
\left\{ \begin{array}{@{}l@{}}
\alpha _{1} = \alpha _{2} + \alpha _{3},\\
\beta _{2} = \beta _{3},\\
\omega _{1} = \omega _{2} + \omega _{3}.
\end{array} \right.
\end{eqnarray*}

The H-type transition can be expressed using the following equations:


(7) \begin{eqnarray*}
\left\{ \begin{array}{@{}l@{}}
f_{1} = \widehat{A_{1}(y)}\exp (\mathrm{i}\alpha x - \mathrm{i}\omega _{1}t) , \\
f_{2} = \widehat{A_{2}(y)}\exp [\mathrm{i}(\alpha /2)x + \mathrm{i}\beta z - \mathrm{i}(\omega _{1}/2)t] ,\\
f_{3} = \widehat{A_{3}(y)}\exp [\mathrm{i}(\alpha /2)x - \mathrm{i}\beta z - \mathrm{i}(\omega _{1}/2)t].
\end{array} \right.
\end{eqnarray*}

The O-type transition is formed by the interaction of a pair of oblique waves $(\omega _1, \pm \beta )$ with identical wave angles but opposite directions. First proposed by Goldstein and Choi [22], it is also referred to oblique-wave transition. Oblique-wave transition is often accompanied by the emergence of distinct three-dimensional waves and streaky structures. The O-type transition can be expressed using the following equations:


(8) \begin{eqnarray*}
\left\{ \begin{array}{@{}l@{}}
f_{1} = \widehat{A_{1}(y)}\exp (\mathrm{i}\alpha x + \mathrm{i}\beta z - \mathrm{i}\omega t),\\
f_{2} = \widehat{A_{2}(y)}\exp (\mathrm{i}\alpha x - \mathrm{i}\beta z - \mathrm{i}\omega t).
\end{array} \right.
\end{eqnarray*}

Bypass transition can be further classified into general-sense bypass transition and narrow-sense bypass transition. General-sense bypass transition refers to boundary-layer transition triggered by any finite-amplitude disturbances, such as jet flows, roughness elements, wake interactions, separation bubble or adverse pressure gradients. Narrow-sense bypass transition specifically refers to transition on a smooth flat plate with zero pressure gradient, induced by decaying isotropic turbulence in the free stream with a turbulence intensity in the range of 0.5%–5%.

**Figure 2.**
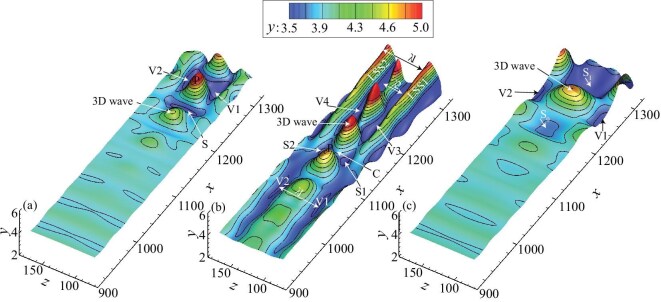
Material surfaces for (a) K-regime transition, (b) O-regime transition, (c) N-regime transition [23]. Reproduced with permission from ref. [23]. Copyright 2020, Cambridge University Press.

Jiang *et al.* [23] visualized the three transition regimes with the deformation of an initially flat material surface, as shown in Fig. 2.

**Figure 3.**
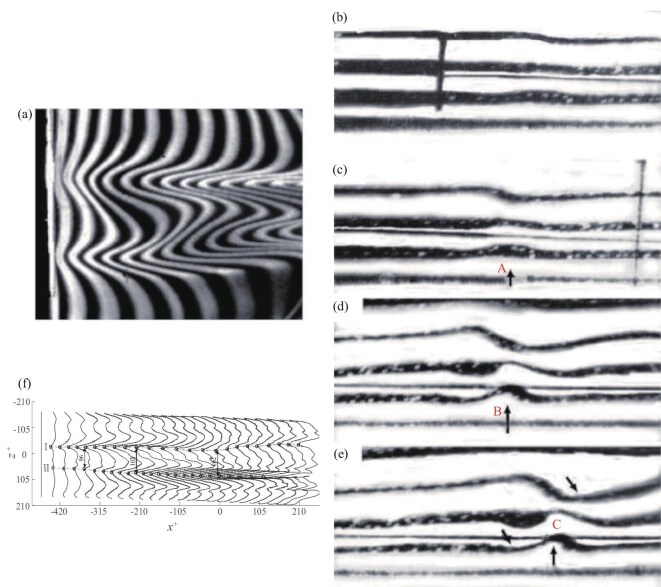
Low-speed streaks in the turbulent boundary layer. (a) Hydrogen-bubble visualization in the plan view [24]. (b–e) Hydrogen-bubble visualization in the side view [13]. Reproduced with permission from ref. [13]. Copyright 1998, Elsevier. (f) A sequence of time lines generated at 50 Hz based on the tomo-PIV database, illustrating low-speed streaks in a turbulent boundary layer. The markers trace the location of the center of two LSSs, designated I and II [24]. Reproduced with permission from ref. [24]. Copyright 2020, Cambridge University Press.

Transition occurs in a water tunnel with a turbulence level of about 1%, where the boundary layer over a flat-plate transitions from laminar to turbulent flow without external control. Since the incoming turbulence level is low, the positions of the low-speed streaks are relatively fixed in the spanwise direction. Figure 3a shows the results of hydrogen-bubble visualization in the plan view, and as we can observe from these results, the flow is naturally blocked at the low-speed streaks. By mass conservation, the fluid should move upward in the wall-normal direction, and the side-view visualization confirms this. Compared to Hama’s classical work [5], we have improved the side-view method by insulating the hydrogen bubbles in segments. This allows for parallel hydrogen-bubble lines in the laminar flow, as shown in Fig. 3b. We placed the hydrogen-bubble lines in the center of the low-speed streaks in the side view, which reveals the wave evolution process. Figure 3(c–e) show this evolution. In Fig. 3c, arrow A indicates that the flow is initially blocked by the low-speed streak and begins to move upward. In Fig. 3d, arrow B represents that the magnitude of the upward movement increases and the flow is blocked more strongly. According to mass conservation, when the flow in the lower layer moves upward, the flow in the upper layer needs to move downward to compensate for the mass. In Fig. 3e, around the upward movement at arrow C, there are downward movements. Jiang *et al.* [24] applied Lagrangian particle tracking based on the tomographic particle-image velocimetry (tomo-PIV) data and extracted the timeline patterns of the low-speed streaks in a turbulent boundary layer, as shown in Fig. 3f, which is qualitatively consistent with the hydrogen-bubble visualization in Fig. 3a.

We conducted systematic experiments on K-type transition, which were carried out in three phases. The first phase was published [17,25]. Both the SCS we discovered and the CS soliton identified by Kachanov were clearly visualized, and we distinguished between the two. As noted by Yaglom and Frisch [26] in their citations on page 553, ‘Lee’s SCS is different from Kachanov’s CS-soliton.’ However, they were concerned about the universal existence of the SCS in shear flows [26].

The second phase of the experiment involved further improvements to the hydrogen-bubble technique, enabling measurements closer to the wall. During this phase, we observed a secondary closed vortex. The SCS underwent primary instability to generate $\Lambda$ vortices (discovered by Hama), while its secondary instability of the SCS led to the formation of the secondary closed vortex [27]. The interaction between the $\Lambda$ vortices and the secondary closed vortex produced Kachanov’s CS solitons, i.e. the chain of ring vortices, as identified by Kachanov. Kachanov proposed that the $\Lambda$ vortices, through self-induced stretching and reconnection, would form a chain of vortex rings [6]. Based on our flow visualization results, however, we proposed that the boundary instability of the SCS generated the $\Lambda$ vortices, and its secondary instability led to the formation of the secondary closed vortex. This secondary closed vortex, interacting with the $\Lambda$ vortices, subsequently formed a chain of ring vortices. We do not dispute Kachanov’s findings; our work presents experimental results directly derived from our observations. These works received substantial positive feedback, particularly from a remarkable summary [28], which recognized our work as one of the classic studies on boundary-layer transition and praised its clarity in addressing several key issues. In 2019, we wrote a review article [2] on flow structures in transitional and turbulent boundary layers at the invitation of the *Physics of Fluids* editor. This review was widely regarded as comprehensive and insightful, ranking among the journal’s top five most-cited reviews. Notably, Chen’s doctoral dissertation from Nanyang Technological University in Singapore [29] replicated our results in great depth for the first time using direct numerical simulation (DNS), offering high praise for our discoveries.

Later, we repeated much of this work for a third phase, particularly using particle-image velocimetry (PIV) technology to replicate the K-type transition experiments. To address Frisch’s question, we also conducted computational studies on N-type and O-type transitions. NPSE analysis and DNS results for K-type and bypass transitions demonstrated that the SCS is the universal structure in N-type, O-type and bypass transitions [23,24,30]. In particular, the 2021 paper was selected for presentation at the IUTAM Special Event: Fluid Mechanics in the Spirit of G. K. Batchelor [31]. This article was granted an exemption from page restrictions because of its importance.

Hu *et al.* [32,33] investigated the evolution of turbulent spots in a flat-plate boundary layer using tomo-PIV and DNS. Both the experimental and numerical results revealed that the SCS plays a pivotal role in turbulent spot development. As the turbulent spot evolves downstream, low-speed streaks emerge at its edges, displaying behavioral characteristics consistent with the SCS. The uplift of these waves drives surrounding fluid toward the wall, inducing vortex accumulation at their boundaries. This process generates high-shear layers at the wave interfaces, which undergo roll-up to form coherent vortical structures. The study confirms that the SCS is central to vortex generation within turbulent spots and significantly influences their evolution. The experimental data of Hu *et al.* illustrate the deformation of a material surface released in a low-speed region within a turbulent spot (see fig. 11 of [32]). The surface reveals elevated streamwise streaks composed of multiple SCSs. This observation underscores that turbulent spots are complex flow entities comprising SCSs and their induced vortices.

In the turbulent boundary layer, the origin of turbulence was first hypothesized by Theodorsen in 1952 [34], who suggested the existence of a two-dimensional vortex tube that, when disturbed and lifted up, forms hairpin vortices in the boundary layer. Later, Kline *et al.* [7] proposed that turbulence is generated by a burst phenomenon observed in their famous experiment. However, for many years, the nature of this burst remained unclear, until 2005, when we discovered that the burst behavior is controlled by the SCS [36]. This discovery is of significant scientific importance, as it connects this widely accepted phenomenon to a tangible flow structure.

Real turbulent boundary-layer experiments must be capable of revealing this mechanism. Unlike the low-speed streaks observed during transition, the low-speed streaks in a turbulent boundary layer exhibit spatiotemporal variability. Fortunately, PIV technology now allows us to track these structures, and we eventually obtained three-dimensional spatiotemporal data of the streaks. The PIV data analysis revealed that the streaks comprise SCSs. These SCSs dominate the turbulence burst phenomenon, directly linking transition and turbulence research [24].

At this point, all possible scenarios in the boundary layer have been studied, and it has been confirmed that the SCS is a common physical structure that governs the generation of turbulence.

To enable fluid dynamics researchers to understand the flow structures presented in this paper—especially for theoretical researchers to comprehend these complex flow patterns and develop structure-based turbulence models—a detailed description of the experimental procedures is necessary. For detailed experimental descriptions of K-type transition, we refer the reader to [17,23,37]. In essence, the K-type transition is entirely consistent with the experiments conducted by Klebanoff *et al.* [14] and Kachanov [6]. The improvements in our work lie in the enhancement of the hydrogen-bubble visualization method, in addition to hot-wire measurements. Using an approach analogous to medical tomography, we placed hydrogen-bubble wires at different heights within the boundary layer, with the starting positions located as close to the wall as possible. This allowed hydrogen-bubble visualization sections to cover the region from the immediate near-wall area to the outer layer of the boundary layer. Since the flow is periodic, interpreting the three-dimensional structures in the flow field using these tomographic images becomes much easier and helps avoid misinterpretations. In addition to this improved hydrogen-bubble method, we employed three-dimensional tomo-PIV to obtain comprehensive spatiotemporal data of the flow field. The flow structures were reconstructed using timeline and material surface methods, with detailed reconstruction methods described by Jiang *et al.* [23,24,30]. The same approach was applied to three-dimensional DNS data to extract realistic physical structures. For experimental descriptions of turbulent boundary layers, we refer the reader to Jiang *et al.* [24].

We will recreate the entire process of K-type transition. Since readers often find hydrogen-bubble visualization images difficult to interpret, when presenting the experimental hydrogen-bubble images, we simultaneously create corresponding animated visualizations based on the experimental results to help readers understand the evolution of flow structures in different transition processes.

**Figure 4.**
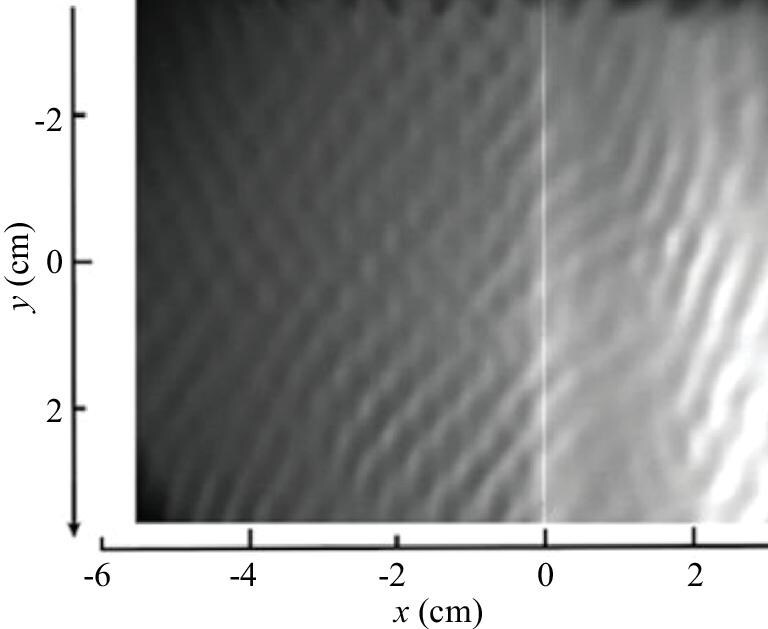
Flow visualization of the flat-plate model leading-edge receptivity process, showing the use of water-surface reflection. Under the disturbance generated at the leading edge (position $x = 0$ cm), the water surface exhibits oblique-wave modes in both upstream and downstream regions. These oblique waves, characterized by stable wavelengths and wave angles, continuously emanate from the leading edge and propagate both upstream and downstream. Oblique waves generate localized three-dimensional waves.

*Step 1.* Two oblique waves interact to generate localized three-dimensional waves, either linear or nonlinear. The linear results are relatively straightforward, whereas we have conducted supplementary experiments on nonlinear interactions. Figure 4 illustrates the interaction of two oblique waves producing localized three-dimensional waves, i.e. the early-stage form of the SCS (see Supplementary data Movie S1).

**Figure 5.**
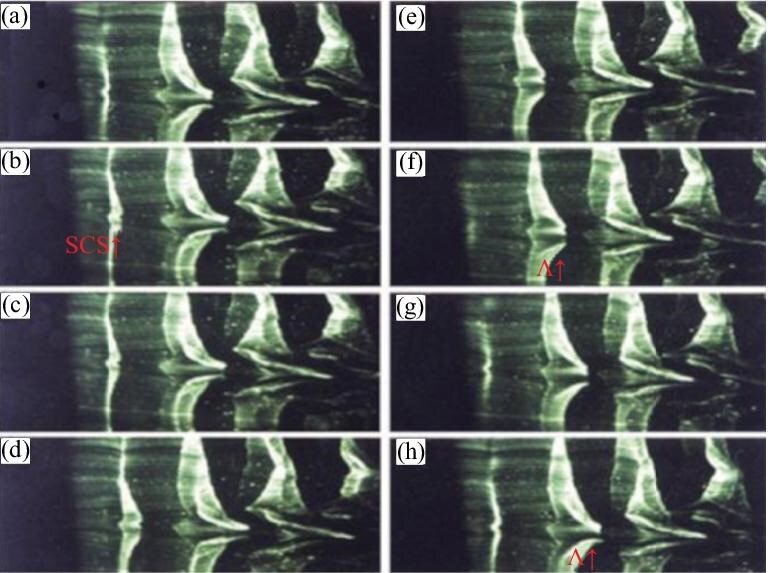
Visualization of the formation of the SCS and the well-known $\Lambda$ vortex. In these pictures, the wire was located parallel to the plate and normal to the flow direction with the flow from left to right in the pictures. The wire was positioned at y = 0.75 mm. The time interval between successive pictures was 1/12 s. (a–f) show the SCS (SCS↑). The formation of the Λ-vortex is clearly visualized in (e–h) (Λ↑) [36]. Reproduced with permission from ref. [36]. Copyright 2005, World Scientific.

*Step 2.* The instability of the SCS leads to the formation of the $\Lambda$ vortex. Figure 5 depicts this process. The underlying mechanism is consistent with our discussion in this paper and in the *Physics Letters A* article (see Movie S2).

**Figure 6.**
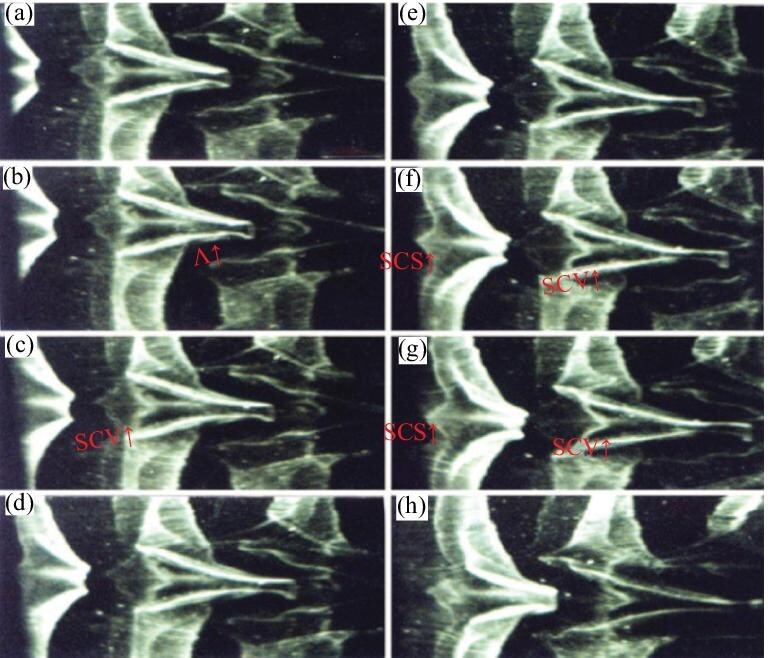
Secondary closed vortex (SCV$\uparrow$) formation. (a–d) Part of the first closed vortex called Λ-vortex, i.e., the Λ-vortex and a SCS appear (SCS↑). (e–h) The right-hand side of the secondary closed vortex appears and then separates from the SCS. At the same time, the Λ-vortex is stretched (Λ↑). The time interval between successive pictures is 1/12 s. This figure is 1/2 of the actual size and the flow is from left to right. The hydrogen-bubble wire was located parallel to the plate and normal to the flow direction. The wire position was x = 300 mm from the leading edge and y = 1.25 mm from the wall [1]. Reproduced with permission from ref. [1]. Copyright 2008, American Society of Mechanical Engineers (ASME).

*Step 3.* The secondary instability of the SCS generates the secondary closed vortex. This process is discussed in detail in Lee and Wu [1], and the results are presented in Fig. 6 (see Movie S3).

**Figure 7.**
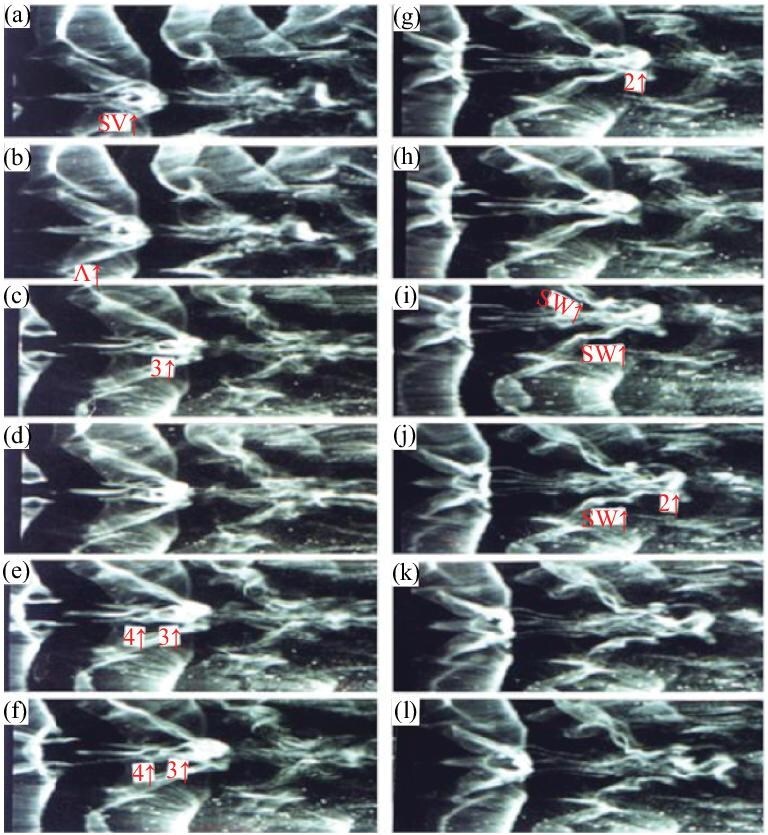
Formation of the second (2$\uparrow$), third (3$\uparrow$) and fourth (4$\uparrow$) ring vortices in a chain of ring vortices. The hydrogen bubble wire was put at x = 350 mm and y = 1.5 mm. The time interval between successive pictures was 1/24 s. The photographs are scaled down to 50% of actual size. (a and b) show that the filaments of the Λ-vortex (Λ↑) are involved in the center of the secondary closed vortex (SV↑) and two symmetric filaments with two narrow necks are formed inside the center of the secondary closed vortex. (c and d) show the breaking and reconnection of the two symmetric filaments in their narrow necks inside the secondary closed vortex and the third ring vortex is clearly presented. In (e) and (f), the fourth ring vortex appears. The filament on its left side comes from the secondary closed vortex and that on its right from the symmetric filaments of the Λ-vortex. (g and l) show the formation of the second ring vortex. The filaments of this vortex come from both the secondary closed vortex and the two symmetric filaments. All three ring vortices appear clearly in (i), (j), (k). Two streamwise vortices (SW↑) appear on the two sides of these three vortices, which are the well-known structures also known as low-speed streaks. The filaments of the streamwise vortex come from both the secondary closed vortex and the Λ-vortex [27]. Reproduced with permission from ref. [27]. Copyright 2001, Springer Nature.

*Step 4.* The interaction between the $\Lambda$ vortex and the secondary closed vortex produces a chain of ring vortices, accompanied by two streamwise vortices on either side of the chain. The chain of ring vortices is the CS solitons identified by Kachanov [6], as shown in Fig. 7 (see Movie S4).

**Figure 8.**
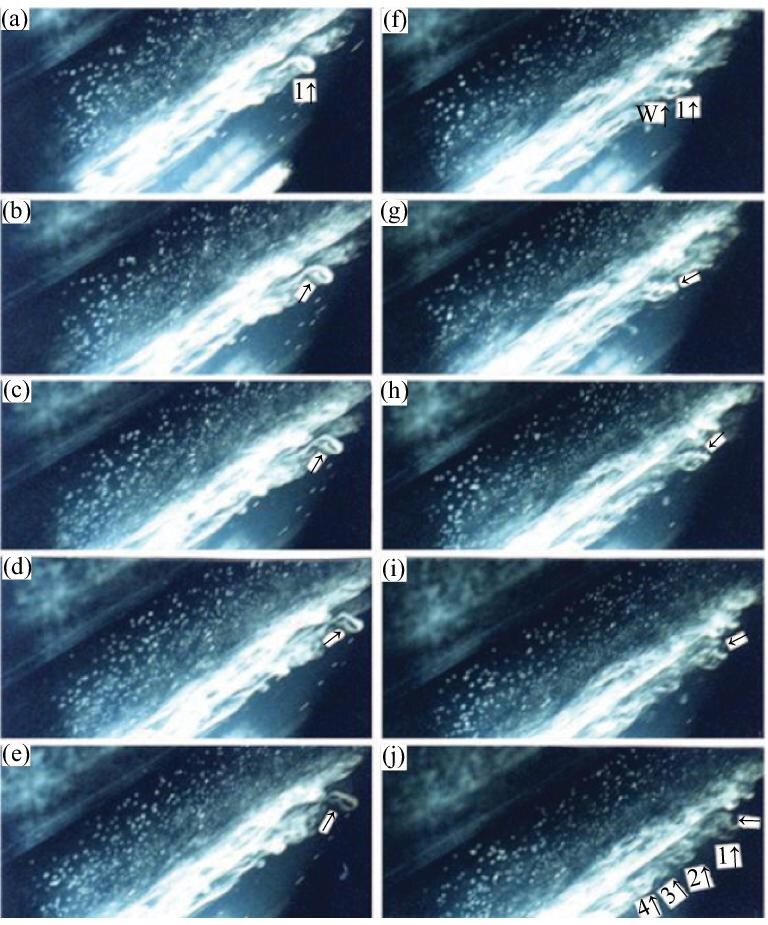
Breakdown of the first ring vortex in a transitional boundary layer. In (a–e), one sees the breakdown of the near-wall part of the vortex. The first ring vortex appears in A. Arrows (↑) indicate the position of the breakdown in the near-wall region of the boundary layer. In (f–j), one sees breakdown of the first ring vortex in the outer region of the boundary layer. (f), (g) The vortex in the outer layer starts to break (1↑), and the vortex in the near-wall region had already broken (W↑). In (h–j), the arrows show the points of the breakdown. In (j), 1$\uparrow$, 2$\uparrow$, 3$\uparrow$, 4$\uparrow$ label the chain of ring vortices [1]. Reproduced with permission from ref. [1]. Copyright 2008, American Society of Mechanical Engineers (ASME).

*Step 5.* The interaction between the chain of ring vortices and the SCS first causes the breakdown of the ring vortices in the near-wall region of the boundary layer, followed by breakdown at the heads of the ring vortices in the outer region of the boundary layer. At this stage, the power spectrum already satisfies the $-5/3$ scaling law, but the dynamics remain a deterministic process, as shown in Fig. 8 (see Movie S5).

## SCS: THE DOMINANT STRUCTURE IN TURBULENCE GENERATION IN SHEAR FLOWS

### Similarity solutions

It is a significant challenge to find solutions to laminar unsteady boundary-layer flows, which are essential for understanding the laminar–turbulent transition. Sun [38] developed a similarity transformation to convert the two-dimensional unsteady laminar boundary-layer equations into a single partial differential equation, and successfully derived similarity solutions for flat-plate boundary-layer flow, expressed in terms of Kummer functions. The superposition of these solutions provides evidence for the existence of the SCS within boundary layers. The finding indicates that all viscous fluid motions have the potential to exhibit the SCS.

### Stratified flow

Jiang *et al.* [39] conducted a stratified inclined duct laboratory experiment in a long pipe with square cross section of dimensions $L=1350$ mm and $H=45$ mm. The exchange flow occurs through a rectangular duct connecting two large reservoirs, which were initially filled with aqueous salt solutions of density $\rho _{0} \pm \Delta \rho /2$. The inclined duct is at an angle $\theta$ relative to the horizontal axis. The Prandtl number is about 700, and the flow was controlled by angle $\theta$ and the Reynolds number $\mathrm{Re} \propto \sqrt{g(\Delta \rho /\rho _{0})H}H/\nu$, where *g* is the acceleration due to gravity. The authors employed a novel laser-sheet-scanning technique, in which PIV and planar laser-induced fluorescence are performed in continuous vertical planes, providing a ‘near-instantaneous’ view of the three-dimensional velocity and density fields.

**Figure 9.**
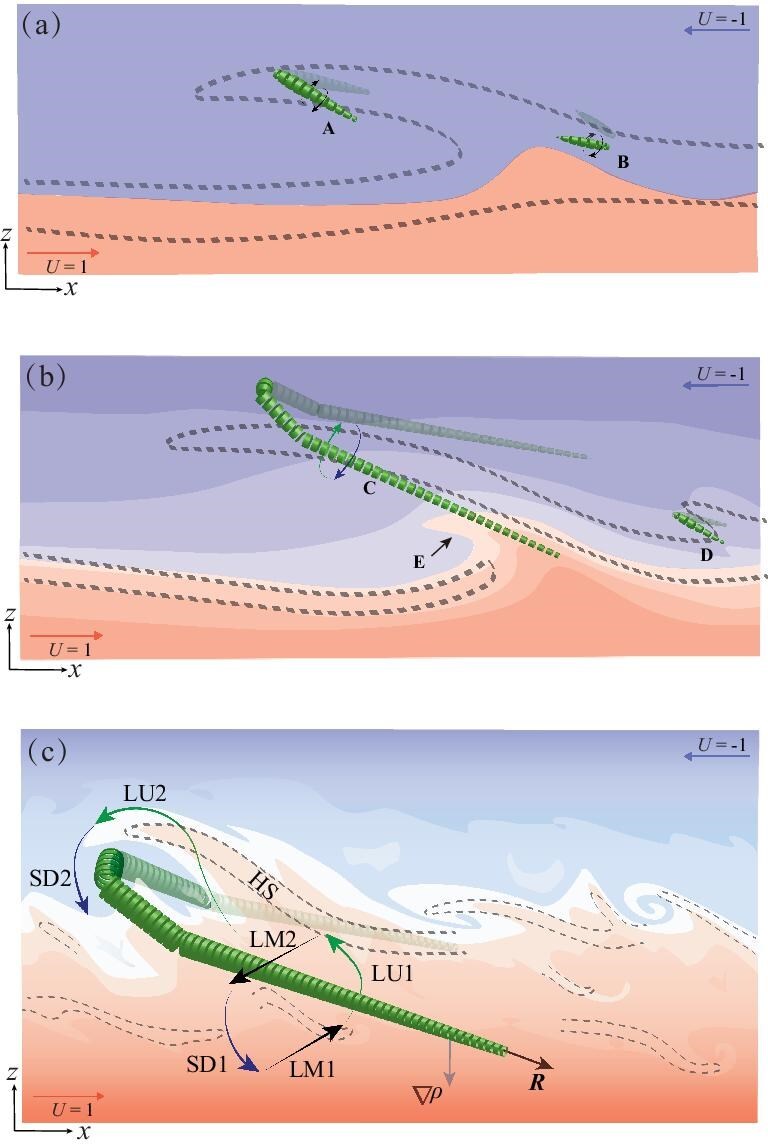
Schematic view of (a) the origin of a hairpin rortex from the Holmboe wave and its evolution in the (b) intermittent regime and (c) turbulent regime. Dashed lines indicate the shear structure, and green segmented tubes indicate rortices (with direction R). The background color indicates density stratification. LU, lift-up; SD, sweep-down; LM, lateral movement; R, rortex vector; HS, high shear [39]. Reproduced under CC BY from ref. [39]. Copyright 2022, Cambridge University Press.

To analyze and visualize vortices, the authors utilized the rortex-shear decomposition method. This approach separates the vorticity field into a rortex vector, representing rigid-body rotation, and a shear vector, facilitating the clear identification of vortex structures, particularly hairpin vortices. Figure 9 illustrates the evolution of the Holmboe wave and its adjacent vortices. The constrained Holmboe instability arises from the resonant interaction between a vorticity wave (at the edge of the shear layer) and a gravity wave (at the density interface). This instability grows to a finite amplitude, enhancing and distorting the background spanwise vorticity until it saturates nonlinearly into the characteristic shear structure of the confined Holmboe wave, as indicated by the dashed line in Fig. 9a. Subsequently, a three-dimensional state with a pronounced vorticity gradient may develop, evidenced by the nucleation of streamwise vorticity on both sides of the wave. These regions are located beneath the wave ‘head’ and above its body. Over time, the vorticity further concentrates at these positions, forming a $\Lambda$-shaped vortex. The newly formed vortices are labeled A and B in Fig. 9a. Figure 9b and c show the schematic models of rortex–density interface interaction in the intermittent regime and the turbulent regime, respectively.

This process is similar to the wave-induced vortex scenario described in boundary layers, where the amplification of three-dimensional Holmboe waves that are the same as soliton-like coherent structures is a key initiator of vortices. This suggests that such structures may represent a universal feature of shear-driven turbulence, regardless of the presence of a boundary.

### Wake flow

Niu *et al.* [40] presented a comprehensive study of the SCS within the wake flow behind a sphere. They investigated the SCS at different Reynolds numbers by large-eddy simulation. The authors used the method named $\widetilde{\Omega _R}$ to identify the vortex structures in the wake flow. It was found that the SCS exhibits similarities to those observed in boundary-layer flows during the turbulent transition.

The authors discussed the simulation results of the wake flow based on different Reynolds numbers.

**Figure 10.**
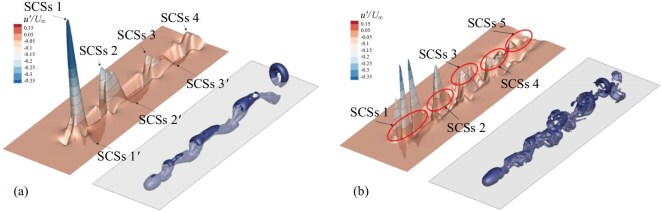
Relative position between the SCS (identified by $u^{\prime }/U_{\infty }$ on $y/d=-0.787$ plane) and the vortex structures (identified by $\widetilde{\Omega _{R}} = 0.23$): (a) ${\rm Re} = 350$, (b) ${\rm Re} = 1000$ [40]. Reproduced with permission from ref. [40]. Copyright 2025, AIP Publishing.

1. *Appearance of the SCS.* At ${\rm Re} = 250$, the structure of wake flow appears as two parallel vortices in the flow direction. At ${\rm Re} = 270$, a ‘kink’ structure forms between the two parallel vortices. Positive disturbances and negative disturbances appear as an alternating pattern. During the ‘kink’ formation stage, the fluctuation amplitude of the flow velocity is about 0.25% of the incident velocity. The amplitudes of velocity fluctuations in the other two directions are even smaller, about 0.07% and 0.06% of the incoming velocity. This indicates that the velocity fluctuations at ${\rm Re} = 270$ already exhibit three-dimensional characteristics.
2. *SCS generation.* At ${\rm Re} = 280$, velocity disturbance in the wake flow generate regular three-dimensional wave packets. Compared with those at ${\rm Re} = 270$, the three-dimensional characteristics of velocity fluctuations under the current ${\rm Re} = 280$ are significantly enhanced. The three-dimensional wave packet moves downstream at about 78% of the incoming velocity. At ${\rm Re} = 300$ and ${\rm Re} = 350$, the three-dimensional wave packets of velocity disturbance appear sharper than those at ${\rm Re} = 280$. In addition, the authors showed the relative positions of hairpin vortices and wave packets with velocity disturbances at different Reynolds numbers, as shown in Fig. 10a. The appearance of the spikes and the stable relative position of the negative wave packet indicate the formation of the SCS.
3. *SCS evolution.* From ${\rm Re} = 350$ to ${\rm Re} = 1000$, the authors found that there is an ordered interaction between velocity disturbance and vortex structure, and their relative positions can be predicted, as shown in Fig. 10b. The wave packet of negative flow-velocity disturbance still maintains regularity. Its shape and amplitude remain almost unchanged in the near-wake region, exhibiting the characteristics of solitary waves. In addition, the authors compared the velocity fluctuations in different directions of the wake flow at different Reynolds numbers (${\rm Re} = 350, 500, 700$ and 1000). The negative peak of *u* is the most significant, followed by *v*, while the magnitude of *w* is relatively small. The difference in time series and amplitude indicates that the negative peak of the *u* component is the key and dominant factor in turbulent transition. As the Reynolds number increases, the fluctuation pattern also changes, from single peak or single valley to multiple peaks or multiple valleys.

In summary, the authors determined that the SCS plays an important role in the wake transition process through numerical simulations. The structure of the wake flow is similar to the phenomenon observed by Lee [1] in his study of boundary laminar flow.

### Falling film

The experiment by Al-Shamaa *et al.* [41] was conducted in a channel with a sinusoidal bottom contour along the flow direction, characterized by an amplitude of 1 mm and a wavelength of 10 mm. The inclination angle $\theta$ of the channel relative to the horizontal plane was adjustable, ranging from 10$^\circ$ to 45$^\circ$ in 5$^\circ$ increments. The working fluid was silicone oil, with the flow rate controlled by a frequency-adjustable pump, allowing control of the Reynolds number.

The three-dimensional patterns of the free surface were identified using the shadowgraph method and light-sheet imaging. In the shadowgraph method, a 400-W halogen lamp illuminated the ceiling, and the reflected light illuminated the free surface. The camera was positioned perpendicular to the inclined bottom of the channel to capture the spatial distribution of the three-dimensional surface patterns along the streamwise and spanwise directions.

**Figure 11.**
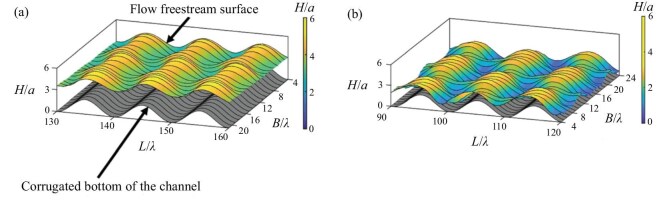
Different three-dimensional patterns detected with the light-sheet technique: (a) synchronous pattern and (b) checkerboard pattern. H: height above bottom troughs; a: amplitude of the bottom contour; $\lambda$: wavelength of the bottom contour; B: distance in the spanwise direction from the sidewall; L: distance from inflow in the streamwise direction. Each line of the free-surface contour corresponds to one profile in a light sheet. Here Re = 39, $\theta = 15^\circ$ (a) and 45$^\circ$ (b) [41]. Reproduced with permission from ref. [41]. Copyright 2023, AIP Publishing.

The amplitude of the three-dimensional patterns was quantitatively measured using the laser-sheet method, which also enabled the determination of the phase shift between the free-surface patterns and the channel-bottom contour. A 450-nm laser was expanded into a laser sheet using a cylindrical lens, with the sheet aligned parallel to the flow direction and perpendicular to the channel bottom. Fluorescent dye quinizarin was added to the silicone oil, and a green color filter was placed in front of the camera to eliminate background laser interference. By moving the laser sheet across the spanwise direction, the entire free surface could be reconstructed, as shown in Fig. 11.

The experimental results reveal that at low Reynolds numbers, the thin-film flow exhibits steady two-dimensional behavior, which transitions to traveling waves and eventually to three-dimensional steady waves. The three-dimensional waves on the free surface consistently emerge near the channel sidewalls, close to the inlet, before expanding to larger regions and reaching the channel center. The three-dimensional patterns can be classified into synchronous patterns and checkerboard patterns. The synchronous patterns have a streamwise wavelength matching the bottom contour of the channel, whereas the checkerboard patterns exhibit surface undulations opposite to the bottom contour. Synchronous patterns appear at all inclination angles, while checkerboard patterns only occur at high Reynolds numbers and large inclination angles.

By decomposing the free-surface profile into Fourier series using the fast Fourier transform for quantitative analysis, it was found that both patterns are triggered only after the free-surface amplitude reaches its maximum. This suggests that these patterns may arise from bifurcations caused by nonlinear resonance of the original mode. The emergence of three-dimensional patterns suppresses traveling waves and stabilizes the steady flow.

### Mixing layer

A spatially developing compressible mixing layer is studied through DNS [42]. The mixing layer, initially laminar, is characterized by a thin layer between two free streams with distinct streamwise velocities but uniform density and pressure. The dimensionless velocities of the upper stream $U_{1}$ and the lower stream $U_{2}$ are set to 1.0 and 0.5, respectively. The Reynolds number and the reference Mach number are specified as 800 and 2.8. Assuming equal specific heats and temperatures yields a convective Mach number $M_c=(\mathrm{Ma}_1-\mathrm{Ma}_2)/2=0.7$. At the inflow boundary, the unperturbed basic inflow profile of the streamwise velocity is obtained by solving compressible boundary-layer equations. In addition, the inflow is forced by three-dimensional unstable waves that are calculated from linear stability theory.

The compressible Navier–Stokes equations are numerically solved using a finite-difference solver. The nonlinear terms are discretized using a fifth-order weighted essentially non-oscillatory scheme. The viscous terms are discretized using a sixth-order central-difference scheme. Temporal advancement of the equations is achieved using a third-order Runge–Kutta method. The computational domain is specified as $[0, 54L_{x}] \times [-L_{y}/2, L_{y}/2] \times [0, 2L_{z}]$, where $L_{x} = 2\pi /\alpha$, $L_{y} = 200$ and $L_{z} = 2\pi /\beta$, with $\alpha$ and $\beta$ representing the most unstable wave numbers from linear stability analysis. The grid sizes are 4160, 350 and 256 along the *x, y* and *z* directions, respectively.

**Figure 12.**
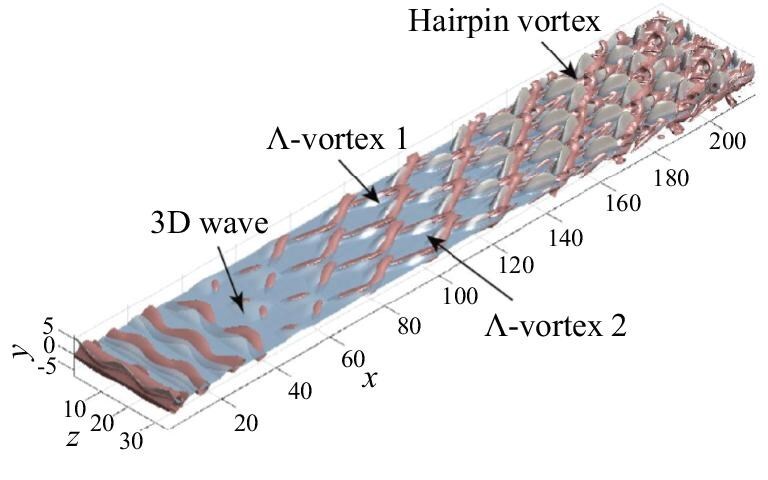
Spatial distribution of the perturbation waves and vortices [42]. Reproduced with permission from ref. [42]. Copyright 2025, AIP Publishing.

Qualitative evidence shows that localized three-dimensional waves travel coherently with vortex structures during the early transition stage. Figure 12 is an oblique view of the three-dimensional flow structures, which displays the growth and metamorphosis of both disturbance waves (represented by positive conditioned normal velocity field *v*) and vortical structures (identified by the second invariant of the velocity gradient) from the inlet to the downstream regions. Initially, the vortex tubes near the inlet boundary are primarily two-dimensional and rapidly become wavy due to the mode resonance of the oblique waves. Then, the wavy spanwise vortex filaments either lift-up or sweep-down, and are stretched into staggered $\Lambda$-shaped vortices as they are convected downstream. The $\Lambda$ vortices further deform into hairpin-shaped vortices and finally break up into smaller-scale structures.

The $\Lambda$-shaped vortices are found to form in the vicinity of high-shear regions, and then stretch into hairpin-shaped structures further downstream. According to statistical results, the formation of streamwise vorticity happens during the stage when the oblique mode remarkably grows, and the normal motion of wave structures causes the surrounding high-shear region. Conditional statistics stress the importance of the local high-shear property in generating enstrophy. It is suggested that generation of hairpin vortices is due to motion of three-dimensional waves along the mean shear flow.

### Jet flow

The wave structures in incompressible circular jets and their relationship to turbulence generation and evolution are investigated with DNS [43], utilizing the OpenFoam open-source software for the solution. By comparing the simulation results with experimental data from the literature, the average velocity distribution along the symmetry axis and velocity distributions at various cross sections are compared, providing preliminary validation of the numerical simulation results, which align with the basic characteristics of circular jets.

**Figure 13.**
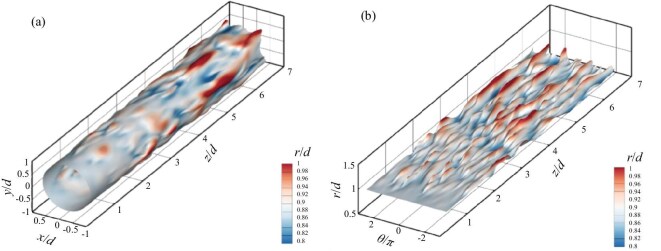
Evolution of the material surface over time: (a) three-dimensional plot, (b) planar projection (using radial displacement for color coding), which clearly shows the generation and propagation of the waves. Reproduced with permission from Associate Professor Q. D. Cai [43], State Key Laboratory for Turbulence and Complex Systems, College of Engineering, Peking University, via personal communication in 2025.

Next, for the three-dimensional flow field obtained from the numerical simulation, Lagrangian material-line and surface-tracking methods are employed to observe the evolution of flow structures. The combination of material-line evolution and three-dimensional velocity field maps reveals that the generation of three-dimensional wave packets is closely linked to the presence of high-speed streaks. Furthermore, through material surface tracking on a full circular pipe surface or local circular arc segment, the entire evolution process of one or more three-dimensional wave packets is depicted in greater detail, as shown in Fig. 13.

For the wave phenomena observed on the Lagrangian material surfaces, the maximum-correlation-point method is used to measure the phase speed of the waves. The measured wave speed is then compared with the local flow velocity. After measuring the wave speed of all three-dimensional waves within the high-speed flow region, it is found that the wave speed is consistently lower than the flow velocity, ranging from 60% to 90% of the flow velocity. This result is consistent with the soliton theory observed in early experiments of boundary layers in flat-plate flows.

Thus, the universally present wave structures in the flow field are closely related to turbulence generation. They exist earlier than vortex structures and play a critical role in the development of the flow. As these wave structures evolve, they lead to the formation of large-scale vortex structures in the flow, ultimately resulting in flow transition to a turbulent state.

### Pipe flow

In the study by Wu *et al.* [44], DNS is performed on a puff of the pipe flow at various Reynolds numbers. Lagrangian approaches are employed to examine the early-stage structure. The formation of low-speed streaks (LSSs) and the lift-up of three-dimensional waves upstream of the puff are observed in a pipe flow using timeline visualizations. The hairpin structure surrounding relatively strong three-dimensional waves is identified using the instantaneous-vorticity-deviation criterion applied to the instantaneous flow field. The formation of vortices consistently follows three-dimensional waves, with a spatial delay determined by the amplitude of the waves. Simulations at various Reynolds numbers are performed to investigate their influence on these physical processes.

For DNS, the unit length is the pipe radius *R*, and the unit velocity *U* is defined as the ratio of the mean volume flow rate to the cross-sectional area of the pipe. Consequently, the unit time is $R/U$. The Reynolds number is based on the pipe diameter $D=2R$ and *U*, with values of 2200, 3000 and 4000. The coordinate is defined as follows: *r* represents the radial coordinate from the pipe axis, *z* denotes the axial coordinate along the flow direction and $\theta$ is the azimuthal coordinate. The finite-difference grid size used in the current computation is $64 \times 192 \times 1151$ along the *r*, $\theta$ and *z* directions for ${\rm Re} = 2200$. For ${\rm Re} = 3000$ and 4000, the grid is $72 \times 216 \times 1152$ in the *r*, $\theta$ and *z* directions, respectively. The dimensionless speed of the upstream front is about 1, 0.82 and 0.684 for ${\rm Re} = 2200$, 3000 and 4000, respectively, and these values are adopted as the moving reference system in the DNS calculations. The incompressible pipe flow solver is the open-source code Openpipeflow30 [45]. Openpipeflow30 employs a second-order predictor-corrector scheme with automatic time-step control for flow simulation in cylindrical domains.

The DNS results are as follows.

**Figure 14.**
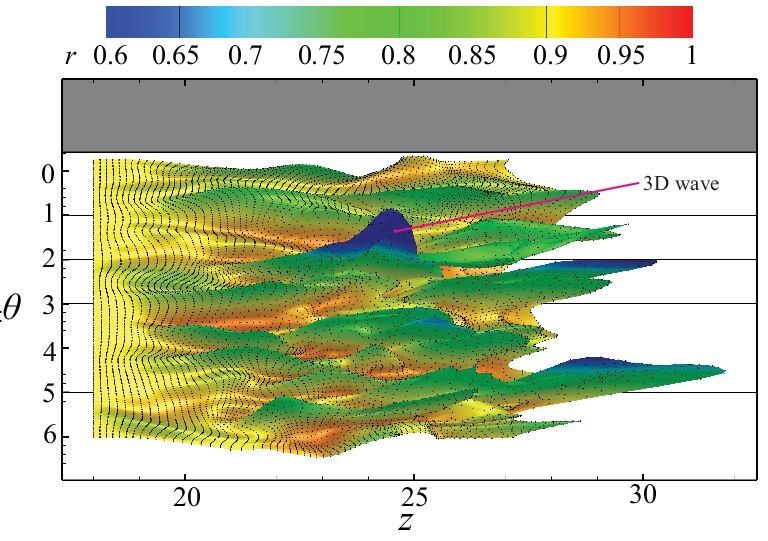
Timelines initiated at $z = 18$, $r = 0.9$ and $\theta = 0 \sim 2\pi$ for ${\rm Re} = 2200$. The ending time is 13.0 [44]. Reproduced with permission from ref. [44]. Copyright 2025, AIP Publishing.

- The gradient of the axial mean velocity profile near the pipe wall increases as Re rises.
- Turbulence generation consistently occurs at the upstream front of a puff or slug in the pipe flow.
- For ${\rm Re} = 2200$, three-dimensional waves are generated during the early stages of the puff, as shown in Fig. 14. These waves are associated with regions of high vorticity. As a three-dimensional wave propagates downstream, vorticity accumulates, leading to the formation of vortices.
- When the amplitude of a three-dimensional wave is sufficiently large and sustained, a high-shear layer forms at its edges. This high-shear layer results in the development of vortices on both sides of the three-dimensional wave.
- The three-dimensional wave lifts away from the near-wall region, and the upward movement of fluid creates a low-speed streak upstream of the three-dimensional wave, observable in most timeline figures, e.g. Fig. 14. Twisting timelines on both sides of the three-dimensional waves. Three-dimensional waves serve as the initial structures responsible for turbulence generation at the upstream front of the pipe flow. Low-speed streaks consist of several three-dimensional waves, which generate high-shear layers and vortices, destabilizing the flow and contributing to turbulence within the pipe. The flow transitions to turbulence more rapidly at higher Re due to the enhanced energy extraction and increased instability. While the patterns of the upstream front vary slightly across different Re values, the fundamental physical process of turbulence generation remains qualitatively consistent.

## FURTHER DISCUSSION ON THE SCS

**Figure 15.**
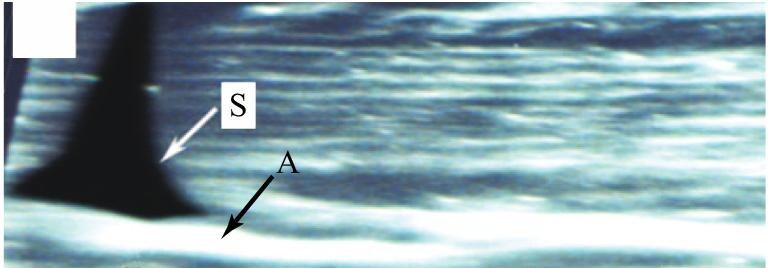
Periodic turbulent bursting in the K-type transition. Reproduced with permission from ref. [1]. Copyright 2008, American Society of Mechanical Engineers (ASME).

Some prominent scholars have long expressed skepticism toward Theodorsen’s hypothesis [34] (see fig. 3 of [35]). A similar physical model was also proposed by Hinze [46], which is widely accepted by most of the fluid mechanics community. The hypothesis fails to account for the formation mechanisms of $\Lambda$ vortices or hairpin vortices. Our three-phase experiments on K-type transition show that the model is incomplete [1,17,23,24,30]. From Hama [5] to Falco [47], Smith [48], Adrian [49] and many others, researchers have sought to elucidate the origins of these vortical structures. These efforts are reviewed in our article [2]. This body of evidence strongly suggests that leading experimentalists remain unconvinced by Theodorsen’s hypothesis. Furthermore, Kline [7] posited that turbulence is generated by bursts in regions of low-speed streaks, independent of the vortex tube (see fig. 10 of [7]). Figure 15 presents the behavior of the SCS during K-type transition, marking the world’s first observation of periodic bursting phenomena (see Supplementary data Movie S6). Arrow S indicates the ejection of the flow, which is the behavior of the SCS. Arrow A indicates the SCS in the form of the streak. This structure is described in detail in our previous publications [1,36,37]. As the SCS moves downstream, hydrogen bubbles within the streak become concentrated. Because of the characteristic ejection motion of the SCS in the vertical plane, the hydrogen bubbles move upward. Since the light sheet illuminates the flow from below, these rising bubbles within the SCS block the light. Consequently, a shadow forms on the upper side of the SCS. These shadows appear periodically in the experimental video recordings. This process represents the bursting of turbulence (see Supplementary data Movie S7).

**Figure 16.**
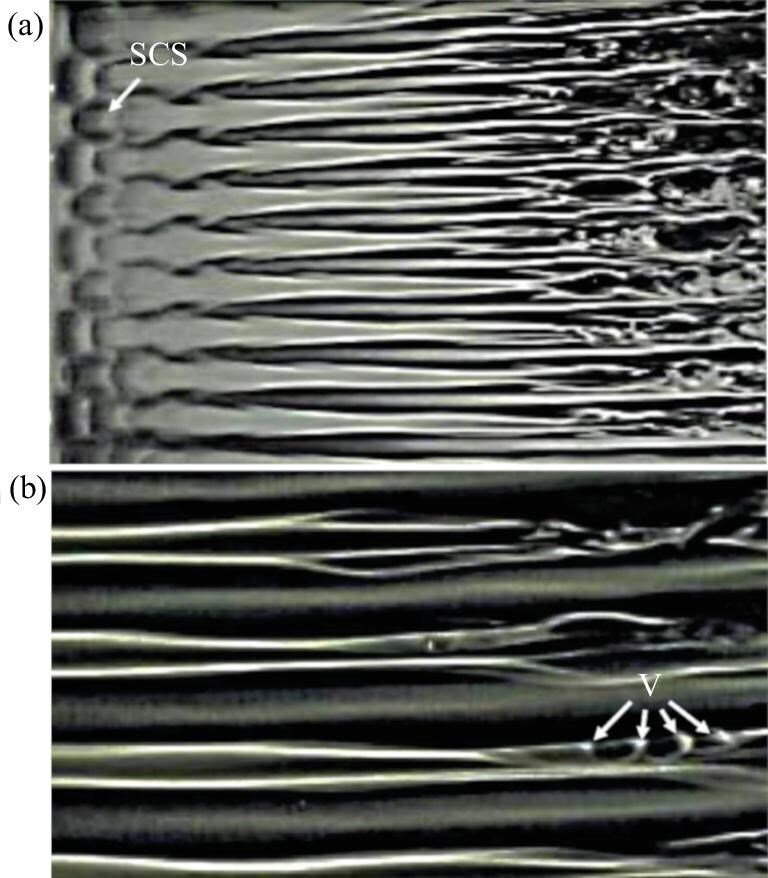
Flow visualization. (a) Several SCSs appear on the left side of each streak (10 streaks in total) [50]. In fact, there are several SCSs inside each streak, and each SCS induces a vortex (on the right side of the streak). The streak formation and the mechanism by which each SCS induces a vortex are the same as those described by Lee and Wu [1]. (b) Detail of (a). Elofsson believed that arrow V indicates the streak breakdown. In fact, arrow V indicates vortices, and each vortex is induced by an SCS rather than by streak breakdown. Reproduced with permission from ref. [50].

Experimental results by leading experimentalists often align more closely with physical reality, while the insights of theoretical and computational experts remain highly commendable. In theories of N-type, K-type and O-type transitions, a fundamental feature is the interaction of two three-dimensional oblique waves. In particular, in the visualization of the O-type transitional experiment by Elofsson [50], the SCS can be clearly seen (Fig. 16). The interactions between two oblique waves inherently generate localized three-dimensional waves, namely, the SCS. However, why have such eminent figures overlooked the existence of the SCS? Theoretical and computational scholars have largely focused on studying streaks while neglecting the critical role of the SCS. Consequently, the notion of turbulent spots, as proposed by Kachanov [6], becomes unnecessary. A review of SCS behavior in various flows reinforces its significance. Hama’s [5] hydrogen-bubble experiments likely revealed its traces, referred to as kink structures. Falco’s [47] ‘pocket vortex’ experiments similarly overlooked the internal structures. Adrian’s [49,51] series of works instead focused on organized vortical structures in a packet pattern and failed to explicitly identify the SCS. Smith and Walker [48] produced highly clear depictions of hairpin vortices and the SCS, yet their focus remained on hairpin vortex formation rather than the SCS. They missed the discovery of the SCS.

The SCS is closely related to the low-speed streak pattern observed near the wall. Lee and Chen [36] presented the streak formation in the near-wall region during K-transition (see fig. 5 of [36]). Several SCSs appear on the left side of the streak. In other words, the streak is composed of several SCSs. In fact, the streak shown in Fig. 16 is the same as Lee’s observation. The streak is also composed of several SCSs.

Wygnanski [52,53] emphasized the importance of oblique waves and their breakdown in turbulence generation, underscoring the role of paired oblique waves. In Poiseuille flow, Carlson *et al.* [54] noted the importance of oblique waves and attributed turbulent-spot formation to oblique-wave breakdown. Their experiments vividly depicted the evolution of multiple streaks, but primarily emphasized oblique-wave breakdown while neglecting the internal structure of streaks. As a result, they missed the opportunity to identify the SCS. Carlson’s experiments, like Wygnanski’s, highlighted oblique-wave breakdown. Currently, while we cannot comment on their intentions, it is important to emphasize that localized three-dimensional waves (the SCS) and oblique waves are fundamentally distinct phenomena. Essentially, the streaks observed in their experiments are consistent with Kline’s [7] streaks. Kline’s remarks on streak importance were unequivocal, and the observed three-dimensional structures from oblique-wave breakdown could reflect the growth of SCS amplitudes within streaks. Simply attributing turbulent spot formation to oblique-wave breakdown oversimplifies the phenomenon. Until Lee [36] demonstrated that streaks are composed of SCSs, the central role of the SCS was largely overlooked. While oblique waves are undeniably significant, the SCS constitutes the primary mechanism of turbulence generation.

This underscores our recent efforts to experimentally validate the generation of the SCS through oblique-wave interactions [8,10]. Although limited experiments have revealed that two oblique waves can produce the SCS, this mechanism remains underexplored. Our 2019 review collated and analyzed literature on the SCS, garnering approximately 150 positive citations, including recognition from some turbulence experts who praised its depth and comprehensiveness [55], even comparing our work to seminal works of Morkovin, Klebanoff, Kachanov and Kline [56–58]. Among overlooked classics is the experimental work of Carlson *et al.* [54] on Poiseuille flow turbulence, which stands as one of the finest depictions of turbulent spot evolution to date. They provided a clear depiction of the evolution of multiple streaks. However, their emphasis on oblique-wave breakdown, coupled with the neglect of streak internal structures, precluded the identification of the SCS.

Let us revisit Hama’s seminal work, as Hama was the first to identify the $\Lambda$-vortex structure—a fact worth reiterating. Hama’s studies, particularly ‘the three-dimensional T–S wave pocket’ [5,59], largely align with the interpretations of Carlson [54] and Wygnanski [52]. These researchers were undoubtedly aware of the importance of three-dimensional waves; however, the waves they described were not localized three-dimensional waves. Crucially, a single three-dimensional oblique wave cannot produce what we define as the SCS. For the localized three-dimensional waves, the concept of ‘localized’ is fundamental to this distinction.

Why do we believe that the SCS better represents the essence of these three-dimensional waves? To answer this, we must reexamine Kline’s definition of bursting, which underscores the SCS as the dominant mechanism driving turbulence bursts. In naturally occurring turbulence, the spatiotemporal characteristics of bursts vary with the conditions of the incoming flow, often manifesting as localized phenomena. Unlike artificially induced transitions, where the SCS aligns uniformly into low-speed streaks, natural turbulence bursts may occur sequentially along the flow direction, forming streaks due to their similar Reynolds numbers. These structures may also appear as isolated events at different spanwise locations and times. Thus, referring to these three-dimensional waves as the ‘SCS’ is more appropriate, as it captures their localized and inherently transient nature.

## NEW DIRECTION OF TURBULENCE RESEARCH

One of the hallmarks of a significant scientific discovery lies in its ability to elucidate phenomena previously unexplained, resolve longstanding challenges or at least provide solutions and insights toward addressing them.

As the saying goes, ‘a craftsman who wishes to do his work well must first sharpen his tools.’ At a minimum, the experimental methods and data-processing techniques we have improved and developed during the course of SCS research offer valuable references for future studies. For instance, the improved hydrogen-bubble method used in Fig. 3(b–e) (side view of the flow field) provides a clearer representation of turbulence bursts. Similarly, the timelines and material surfaces we have developed and utilized allow for the distinction between wave and vortex structures. These advancements represent some of the most critical aspects of fluid mechanics research, where even minor methodological adjustments can open up entirely new frontiers in the field.

Based on our current understanding of turbulence phenomena, particularly shear turbulence, it can be asserted that the SCS serves as the foundational building block for other quasi-coherent structures. Evidently, investigating composite quasi-coherent structures appears to be an unwise endeavor for several reasons, as it unnecessarily complicates research. The study of streak stability serves as an example of focusing on the intricate problem itself rather than on individual structures. Internationally, significant efforts have been devoted to this topic, but mathematically describing a single streak is already a challenge. Investigations into streak spanwise waving or streak breakdown further escalate the complexity of research. Studying streak breakdown is inherently more challenging than directly examining the stability of the SCS itself. Moreover, focusing on streak stability veers away from the physical essence of the problem while significantly increasing the difficulty, often leading to erroneous conclusions. Streak breakdown, as it is commonly referred to, is nothing more than a misnomer. For example, in Fig. 16b, what the original authors described as ‘streak breakdown’ is, in reality, a clear depiction of vortex formation. The arrows labeled ‘V’ in Fig. 16b point to four distinct vortices, each formed by the instability at the boundary of individual SCS within the streak. Let us interpret this process of vortex formation: streaks are composed of SCSs, whose amplitudes grow and then diminish. During this transition, vortices emerge, with most of the dyed material being drawn into the vortex cores, manifesting as discrete vortices while the streak itself dissipates. This mechanism is inherently simple and transparent. However, describing streak instability using the misleading term ‘breakdown’ is fundamentally incorrect. Turbulence research is rife with efforts that yield little return, often characterized by these unnecessary complexities. Naturally, delving into even more intricate composite structures becomes impractical. For instance, attempting to study the dynamic relationships of the complex structures described by Adrian *et al.* [49,51] is evidently beyond reach. Similarly, investigating very-large-scale-motion dynamics remains an unattainable aspiration.

It is imperative to explore new perspectives. For example, Kachanov *et al.* [60] noted the concept of transition ‘without the formation of turbulent spots’, and these profound statements can be succinctly explained if the SCS is understood as the primary driver of turbulence generation.

The formation of turbulent spots is not essential for the onset of turbulence. In water tunnels or wind tunnels with low freestream-turbulence level, K-type transition typically involves the interaction of a fundamental wave with two three-dimensional oblique waves, leading to the formation of the SCS. The instability of the SCS gives rise to $\Lambda$ vortices, while secondary instability of the SCS results in the secondary closed vortex. The interplay between $\Lambda$ vortices and the secondary closed vortex generates the chain of ring vortices, whose subsequent breakup and dissipation lead to turbulence—without any intermediate formation of turbulent spots. Thus, turbulent spots are not necessary for the initiation of turbulence. As illustrated in fig. 5 of [36], only two independent turbulent production channels are present, each completing the entire process of turbulence generation without the emergence of turbulent spots. In flow fields with a higher freestream-turbulence level, more turbulent production channels arise, distributed across various spanwise locations. The temporal and spatial overlap of SCS formation in these channels, i.e. especially those originating early at the spanwise center near the leading edge, facilitates the emergence of vortices downstream. The superposition of the SCS and the related vortices results in the formation of turbulent spots, as elaborated in our recent publications [32,33].

Recently, Spanish scholar Jiménez [61] demonstrated that ‘streaks are not required for the maintenance of wall-bounded turbulence.’ Streaks are also not a necessary component in turbulence generation. To understand this, it is essential to revisit how streaks are formed. In controlled transition scenarios, where the imposed conditions are periodic, SCSs are typically generated periodically at the same spanwise locations, leading to the formation of streaks (Fig. 16a and b). In contrast, during natural transition or within turbulent boundary layers, the generation of the SCS depends on initial conditions. If the initial conditions are analogous to those in controlled transitions, multiple instances of the SCS may be produced at adjacent moments in the same spanwise location, resulting in the formation of streaks. However, if only a single SCS is generated at a given location, it evolves independently to produce turbulence, without forming a streak.

Let us consider another issue: some scholars are deeply invested in studying the interface of turbulence and non-turbulence in the outer layer of turbulent boundary layers, which, in essence, is also a misnomer. Turbulence itself is inherently unsteady, characterized by the rise and fall of different structures over time and space. The spatiotemporal variability of its boundary is, fundamentally, a straightforward phenomenon. Yet, it is perplexing that significant time and effort have been spent investigating this matter. Essentially, the generation of the SCS depends on initial conditions, and where the SCS arises, the boundary-layer thickness evolves and convects downstream with it. This problem, if it even exists, is a minor one.

Drag reduction is a longstanding theme. However, the issue of turbulent drag—particularly turbulent skin friction—deserves more attention. Research efforts aimed at reducing drag in boundary layers have become an enduring pursuit, with generations of researchers following in one another’s footsteps. However, without a clear understanding of the mechanisms underlying turbulent drag formation, much of this research has been aimless. While there are a few successful cases, most studies have been inconclusive. Fundamentally, the primary contributor to turbulent drag is the SCS. As illustrated in Fig. 3a, the SCS obstructs flow, and its contribution to skin friction is qualitatively significant. From the perspective of its convection velocity, the SCS convects downstream at approximately 0.6 to 0.8 times the freestream velocity, occupying the entire bottom layer of the boundary layer. During the evolution of the SCS in this region, from a laminar velocity profile to a turbulent one, it contributes significantly to drag. This is because the bottom layer of the boundary layer contains no other flow structures; the SCS alone dominates it. Therefore, the SCS is the principal contributor to turbulent skin friction. To reduce drag, it is clear that modification of the SCS properties is required. This is the core of drag reduction, and it explains why progress in reducing turbulent skin friction has been so limited. The intrinsic soliton wave nature of the SCS makes altering its properties a physically challenging task.

The issue of turbulence remains unavoidable. Fundamentally, turbulence is not a well-defined problem, as it lacks a precise definition; therefore, the ‘solution to turbulence’ is itself a misnomer. However, by concretizing the turbulence problem, we can approach it more meaningfully. Specifically, we focus on the generation and development of boundary-layer turbulence. This discussion can be divided into three levels: comprehension of the physical process, theoretical prediction, and strategies for control and utilization.

The first level involves understanding the generation and development of boundary-layer turbulence. Using current theoretical calculations and experimental methods, it is possible to achieve a comprehensive understanding of these processes. Existing DNS effectively capture the major processes involved in the generation and development of boundary-layer turbulence. In this sense, the first-level problem, i.e. the process of boundary-layer turbulence generation and development, can be resolved. Similarly, for other specific flow-related problems, current methodologies can provide solutions. The second and third levels, i.e. predictive theories and the control and utilization of turbulence, are, in principle, achievable in the foreseeable future.

It is worth noting that, in 2004, traveling waves were discovered in a pipe flow [62], which are essentially the same physical structure as the SCS. Since 2004, the term ‘traveling wave’ has also been used in boundary-layer research, though this discussion lies beyond the scope here. The primary reason is that we discovered the SCS nine years earlier than the identification of traveling waves, and our discovery established a self-contained system.

Nagata [63] was the first to obtain finite-amplitude traveling-wave solutions in plane Couette flow. Some time after our discovery of the SCS, Waleffe [64] also derived traveling-wave solutions in Couette flow. Later, these were referred to as exact coherent structures in pipe flow [65] and were considered experimentally confirmed in pipe flow by Hof *et al.* [62]. Traveling waves and exact coherent structures are composite forms of several structures. Judging from their visualizations and mathematical expressions, they are composite structures composed of several vortices and streaks. While it is undeniable that they have contributed to turbulence research, as we pointed out in our article [2], composite structures do not reveal the dynamics as clearly as fundamental structures like the SCS. They also refer to traveling waves as the building blocks of turbulence generation, but we believe that the SCS is more fundamental and better explains the physical essence of turbulence generation, such as revealing the dynamical processes of turbulence bursting and low-speed streak formation.

## Supplementary Material

nwaf535_Supplemental_Files
